# Composition Based Strategies for Controlling Radii in Lipid Nanotubes

**DOI:** 10.1371/journal.pone.0081293

**Published:** 2014-01-02

**Authors:** Michael E. Kurczy, Lisa J. Mellander, Neda Najafinobar, Ann-Sofie Cans

**Affiliations:** 1 Department of Chemical and Biological Engineering, Chalmers University of Technology, Gothenburg, Sweden; 2 Department of Chemistry and Molecular Biology, University of Gothenburg, Gothenburg, Sweden; University of Cambridge, United Kingdom

## Abstract

Nature routinely carries out small-scale chemistry within lipid bound cells and organelles. Liposome–lipid nanotube networks are being developed by many researchers in attempt to imitate these membrane enclosed environments, with the goal to perform small-scale chemical studies. These systems are well characterized in terms of the diameter of the giant unilamellar vesicles they are constructed from and the length of the nanotubes connecting them. Here we evaluate two methods based on intrinsic curvature for adjusting the diameter of the nanotube, an aspect of the network that has not previously been controllable. This was done by altering the lipid composition of the network membrane with two different approaches. In the first, the composition of the membrane was altered via lipid incubation of exogenous lipids; either with the addition of the low intrinsic curvature lipid soy phosphatidylcholine (soy-PC) or the high intrinsic curvature lipid soy phosphatidylethanolamine (soy-PE). In the second approach, exogenous lipids were added to the total lipid composition during liposome formation. Here we show that for both lipid augmentation methods, we observed a decrease in nanotube diameter following soy-PE additions but no significant change in size following the addition of soy-PC. Our results demonstrate that the effect of soy-PE on nanotube diameter is independent of the method of addition and suggests that high curvature soy-PE molecules facilitate tube membrane curvature.

## Introduction

Lipid vesicle-nanotube networks are a promising platform for small-scale chemical studies, including cellular transport phenomenon [1], [2], vesicular content release through exocytosis [3], [4] and carrying out confined chemical reactions [4]. These incredibly versatile and flexible structures are well characterized and the size of the vesicles and the length of the nanotubes connecting them is highly controllable [5]. However, the diameter of the nanotube is not easily manipulated and until recently, difficult to measure [6], [7]. A predictable method for tailoring the diameter of the nanotube connectors will increase the utility of these structures, by for example adding the ability to control diffusion limited flux or presenting the possibly to carry out size exclusion separations. Additionally, this would be an important parameter to control if using this system as an artificial cell mimic to study e.g. transport and lipid sorting in highly curved membranes during intracellular or extracellular nanotube transport.

**Figure 1:**
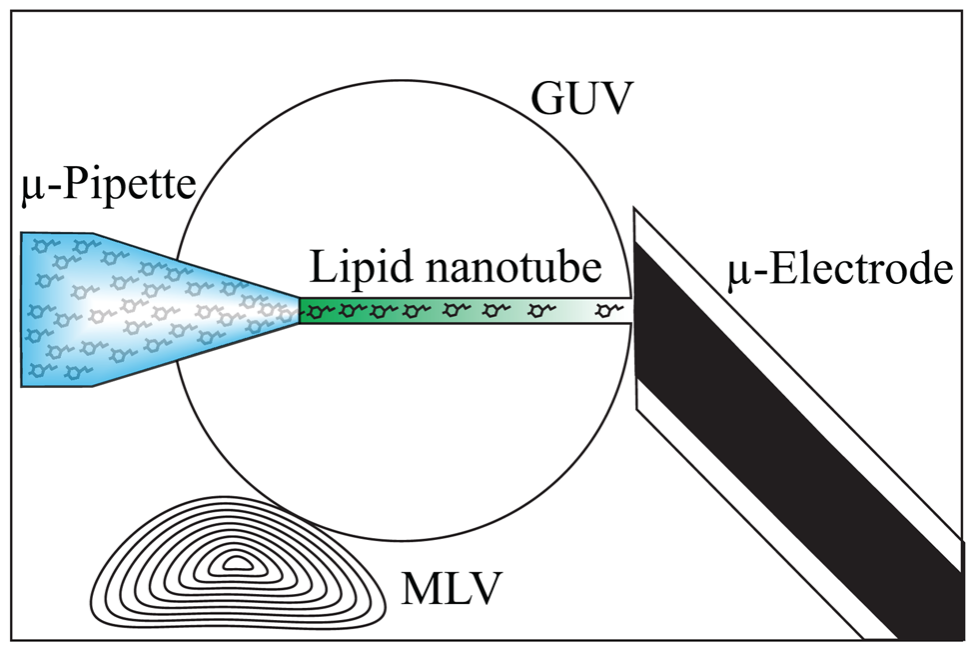
Experimental set up. A giant unilamellar vesicle (GUV) is formed from a multilamellar vesicle (MLV) that is attached to the glass slide substrate. A micropipette filled with dopamine is inserted into the GUV and through the second membrane through electroporation. The pipette is then pulled back into the vesicle bringing with it a nanotube connecting the pipette to the outside of the vesicle. At the exit of the tube a micro electrode is positioned and the current through the nanotube is monitored with zero pressure applied through the micropipette.

In this work lipid nanotube radii were measured using the experimental set up shown in the schematic in Figure 1 and described previously [6]. A glass micropipette, back-filled with a solution of an electroactive molecule with a known diffusion coefficient, was used to pull a nanotube into the interior of a giant unilamellar vesicle (GUV). A multilamellar vesicle (MLV) attached to the GUV provides excess lipid material to facilitate nanotube formation under low tension. A carbon fiber microelectrode was placed in close proximity to the GUV membrane and positioned at the exit of the lipid nanotube. The microelectrode was used to electrochemically monitor the current generated by the oxidation of the electroactive molecules as they exit the nanotube. The diffusing molecules were continuously oxidized at the electrode surface creating a permanent concentration gradient between the tip of the pipette and the electrode. A model of diffusion-based transport was implemented to relate the steady-state amperometric current to the flux of electroactive molecules at the nanotube opening. The measurements of the steady-state current together with simultaneous measurements of the lipid nanotube length were used to calculate the nanotube diameter (equation 1). This method has afforded the ability to track nanotube diameter with respect to changes in lipid composition without the use of labels, which might convolute the result by contributing to the measured effect.

The spontaneous curvature of a membrane is, in broad terms, the curvature that the bilayer would assume if it was unrestrained [8]. This property is closely related to the intrinsic curvature [9] of the membrane components. Depending on their molecular geometry, phospholipids can be described as having a positive, negative, or neutral influence on the spontaneous curvature of the lipid monolayer. This classification is based on the cross-sectional area of the hydrophilic head group as compared to the hydrophobic acyl chain tail group. In general, the sum intrinsic curvature of the individual membrane components is a good approximation of the spontaneous curvature of a lipid monolayer. However, because the two monolayers are strongly coupled, the net curvature of a symmetric bilayer is zero. Thus, for an intrinsically curved lipid to affect the spontaneous curvature of a bilayer it must be asymmetrically distributed. Indeed, the ability to create asymmetric bilayers is critical to composition based lipid nanotube diameter manipulation.

**Figure 2:**
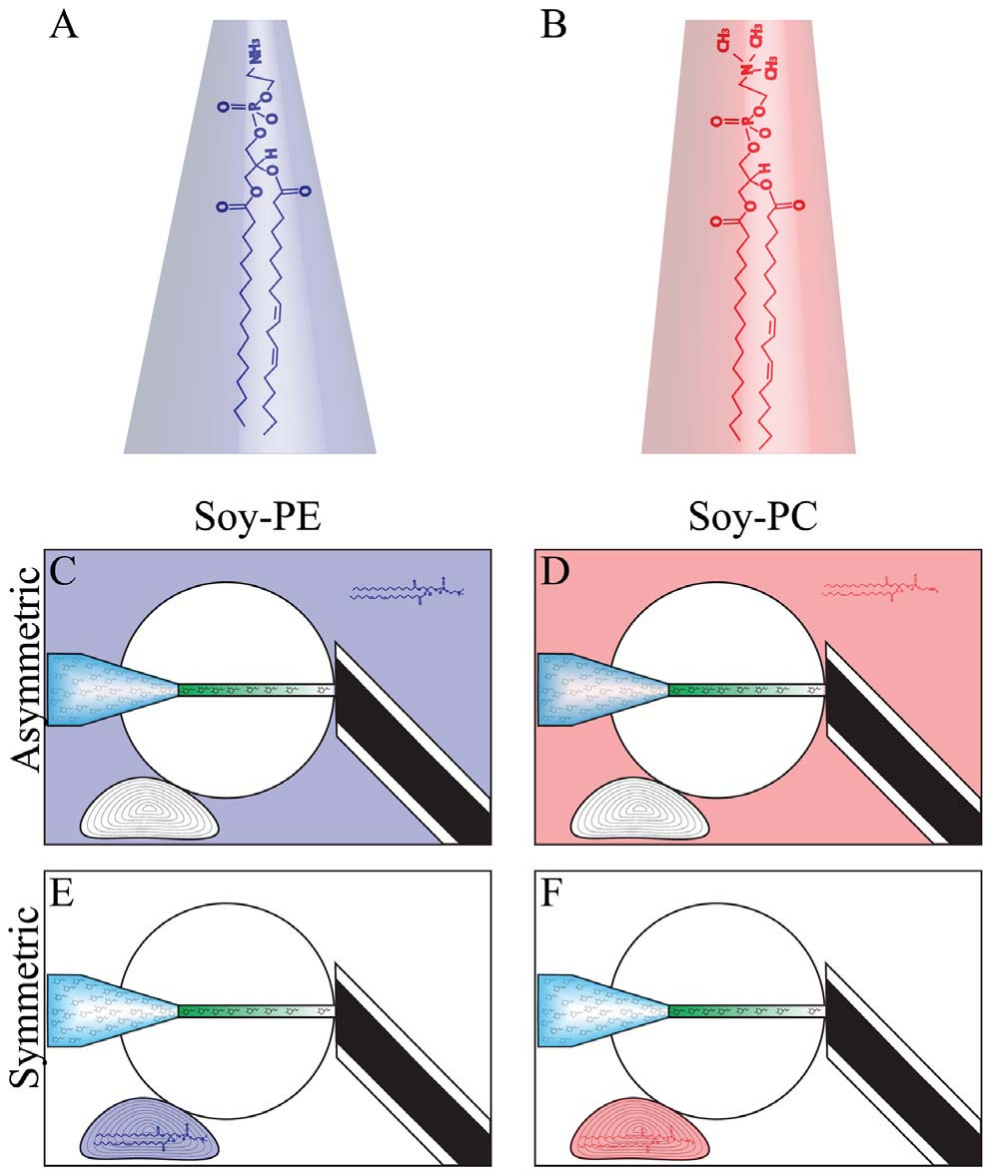
Methods for altering the lipid composition of the GUV. The GUV was enhanced with respect to the lipids PE and PC. Two methods of enhancement were applied, an asymmetric enhancement through lipid incubation of the outer lipid leaflet of the vesicle membrane and a symmetric enhancement which was accomplished by changing the lipid composition of the starting material during GUV formation and therefore also the MLV.

We have evaluated two strategies (Figure 2), based on intrinsic curvature, to control the inner diameter of lipid nanotubes pulled from a soy bean lipid extract (SBL) vesicle-nanotube assembly [3]. The first approach taken was to induce lipid asymmetry by incubating one leaflet of the membrane with an exogenous lipid to directly affect the spontaneous curvature of the membrane. In the second approach we added exogenous lipid to the total lipid composition during liposome formation, aiming to affect the spontaneous curvature via curvature-coupling, the notion that lipids and proteins may locally reorganize to facilitate curved structures [10]. Assuming that curvature-coupling applies to this system, it follows that increasing or decreasing the availability of intrinsically curved lipids should lead to a change in lipid nanotube diameter. In this study soy phosphatidylethanolamine (soy-PE) was used to increase the spontaneous curvature of the lipid membrane and soy phosphatidylcholine (soy-PC) was used to decrease the curvature. Following each lipid incubation treatment, lipid nanotube sizes were measured and compared to measurements made at untreated nanotubes.

In general we have found that independent of lipid enhancement methods, the addition of soy-PC to the SBL mixture had a minimal effect on tube diameter while addition of soy-PE caused the tube to constrict. Thus it seems that the total availability of lipids with intrinsic curvature determines the tube diameter. The tube diameter can therefore be controlled by either changing the ratios of the curved elements in the starting material, or incubation can be used to dynamically change the tube diameter.

## Materials and Methods

### Chemicals and materials

The rehydration buffer for the liposome preparation was prepared with 5 mM Trizma base, 15 mM K_3_PO_4_, 30 mM KH_2_PO_4_, 15 mM K_2_HPO_4_, 0.5 mM EDTA and 1 mM MgSO_4_ at pH 7.4. Lipids were obtained from Avanti Polar Lipids, Alabaster, AL. All other chemicals were of analytical grade and purchased from Sigma-Aldrich (Sweden) and used as received.

### Electrode fabrication and electrochemical data acquisition

Carbon fiber micro electrodes were prepared by aspirating single 5-µm diameter carbon fibers into borosilicate glass capillaries (1.2 mm O.D., 0.69 mm I.D., Sutter Instrument Co., Novato, CA). The filled capillaries were then pulled using a commercial micropipette puller (Model PE-21, Narishige, Inc., London, UK) and the electrodes were sealed with epoxy (Epoxy Technology, Billerica, MA). The electrode tips were polished at a 45° angle on a diamond dust-embedded micropipette beveling wheel (Model BV-10, Sutter Instrument Co., Novato, CA). Electrodes were tested in a solution of 100 µM dopamine before experiments and only electrodes with stable I-E curves were used. For measurements, the working electrode was placed against the liposome-nanotube junction using a piezo-micropositioner (PCS-750/1000, Burleigh Instruments, Fishers, NY) and held at +800 mV versus a silver/silver chloride reference electrode (Scanbur, Sweden) using an Axon 200B potentiostat (Molecular Devices, Sunnyvale, CA). The output was digitized at 5 kHz and filtered at 2 kHz via an internal four-pole low-pass Bessel filter. Further filtering of the output was applied via a 10 Hz low-pass digital filter within the AxoScope 10.2 software (Molecular Devices, Sunnyvale, CA) prior to analysis using MiniAnalysis software (Synaptosoft, Inc., Decatur, GA).

### Liposome preparations and manipulations

Surface-immobilized giant unilamellar soybean liposomes (SBL) were prepared from soybean polar lipid extract, as previously described [3], [5]. Briefly, the lipid extract dissolved in chloroform was dried using a rotation evaporator (Büchi, Switzerland). The dried lipid film was rehydrated with rehydration buffer at 4°C for 24 h and then sonicated on ice for 15 min. 3 µL of the lipid suspension was then micropipetted onto a glass coverslip (Menzel-Gläser, Braunschweig, Germany; 24 mm×60 mm, no. 1) and dehydrated in a vacuum desiccator. The slides were transferred to the microscope for experiments and the film was again hydrated using rehydration buffer. This method produces unilamellar liposomes attached to a multilamellar liposome that can operate as a lipid reservoir during the experiment. Augmentations of the SBL preparation were made with soy-PC or soy-PE at 10, 20 and 30% by total weight.

Liposome experiments were performed as previously described [6]. Briefly, injection pipettes were pulled from borosilicate glass capillaries (1.0 mm O.D., 0.78 mm I.D., Sutter Instrument Co., Novato, CA), using a commercial pipette puller (Model PE-21, Narishige Inc., London, UK). A pipette, back-filled with 50 mM dopamine dissolved in rehydration buffer, was placed against the liposome membrane. A counter electrode for electroporation, constructed from a pre-fabricated 5-µm carbon fiber tip (ProCFE from Dagan Corp, Minneapolis, MN) was placed on the opposite side of the liposome from the injection pipette. The pipette was then electro-inserted into the liposome with the aid of a 4 ms, 40–60 V voltage pulse generated by a constant voltage isolated stimulator (DS2A-Mk. II, Digitimer, Inc., Hertfordshire, UK). The dopamine solution was injected into the unilamellar liposome by means of a Femtojet microinjector (Eppendorf/Brinkmann Instruments, Hauppauge, NY), increasing its size through inflation. Once liposomes were at a workable size (>50 µm in diameter), they were further manipulated by puncturing the second wall of the liposome from inside and out, with the injection pipette and allowing the liposome membrane to seal around the pipette tip. The pipette was then slowly retracted into the interior of the vesicle, pulling a lipid nanotube attached between the liposome and the injection pipette.

For the experiments where lipid incubations were performed on the outside of the liposome membrane, the lipid nanotube size was initially measured at the liposome with non-altered lipid composition. Thereafter, the exterior was incubated by the addition of lipid solution into the liposome hydration media. The concentration of the final lipid incubation solution was 100 µM. The lipids used for the augmentations, were soy-PC and soy-PE. Following 30 min of lipid incubation a second new lipid nanotube was pulled, and the size of the lipid-augmented nanotube was measured.

### Nanotube radius measurements and calculations

The flux of dopamine through the nanotube was measured using carbon fiber amperometry. A 5-µm carbon fiber microelectrode was placed at the nanotube-liposome junction and when a stable current level was reached, the steady-state current was recorded for ∼30 s. The electrode was then removed from the nanotube-liposome junction and allowed to reach a steady current in the absence of electroactive species to establish a baseline. The difference in measured current for a nanotube versus this background, together with the length of the nanotube as determined from simultaneous DIC imaging, was then used to compute the diameter of the nanotube based on the previously derived relationship (equation 1) where r is the radius of the nanotube of a given length (*L*), Δi is the change in measured current with respect to the background, *n* is the number of moles of electrons transferred per mole of redox species (for dopamine, this is equal to 2), *F* is Faraday's constant (96,485 coulombs/mole of electrons), *D* is the diffusion coefficient of the selected redox species (for dopamine this is equivalent to 6.7×10^−6^ cm^2^·s^−1^ in buffer solution) [11]. ΔC is the change in concentration of dopamine over the nanotube length and is equal to the concentration of electroactive species in the pipette assuming that the concentration at the electrode surface is zero.
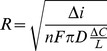
(1)


### Microscopy and Digital Video Recording

Experiments were monitored using an Olympus IX-71 microscope (Olympus, Melville, NY) with a 40× oil objective (Olympus, UApo/340 40× oil iris, NA 1.35). Differential interference contrast microscopy (DIC) was utilized for a pseudo-three dimensional appearance of the liposome. An Olympus SC20 digital color camera interfaced to a personal computer with Cell-A software (Olympus, Hamburg, Germany) was used for visual recording of the experiments. Nanotube lengths were measured from digital images captured during electrochemical measurements via the Cell-A software interface.

## Results

Manipulation of lipid nanotube diameter based on spontaneous curvature can be rationalized by considering the Helfrich bending energy of a lipid nanotube at equilibrium (equation 2) [8], where *K* is the bending rigidity of the membrane, *H* is the membrane mean curvature, *C_0_* is the spontaneous curvature, *A* is the membrane surface area, σ is the surface tension of the membrane, *p* is the interior pressure of the volume (*V*) enclosed by the membrane, and *f* is the point force necessary to pull and sustain the membrane a distance *L*. The equation shows that the surface area of the membrane and therefore the radius of the nanotube depends on the relationship between the bending rigidity and the surface tension. It is clear that the spontaneous curvature will affect the magnitude of *K* resulting in an effective bending rigidity (*K_eff_*). Equation 3 is used to calculate the radius (*R*) of the nanotube and shows that the change in *K_eff_* will be reflected in the measured radius and that the magnitude of the change will be attenuated by the degree of tension in the system.
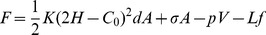
(2)

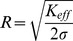
(3)


The first strategy investigated to control nanotube diameter was to manipulate the effective bending rigidity of the membrane by actively inducing an asymmetric bilayer. Since the spontaneous curvature of a bilayer is the sum of the monolayer spontaneous curvatures, intrinsic curvature must be added asymmetrically to make a net increase or decrease. Asymmetric lipid compositions in the GUV membranes were created using an incubation scheme as illustrated in Figure 2 C & D. The GUV is a sealed system, allowing the addition of exogenous lipid to only affect the distal monolayer. This method lacks control in that it is difficult to predict the amount of lipid that will be incorporated, but it has the advantage of being dynamic, that is to say the diameter can be changed in real time through lipid incubation.

**Figure 3:**
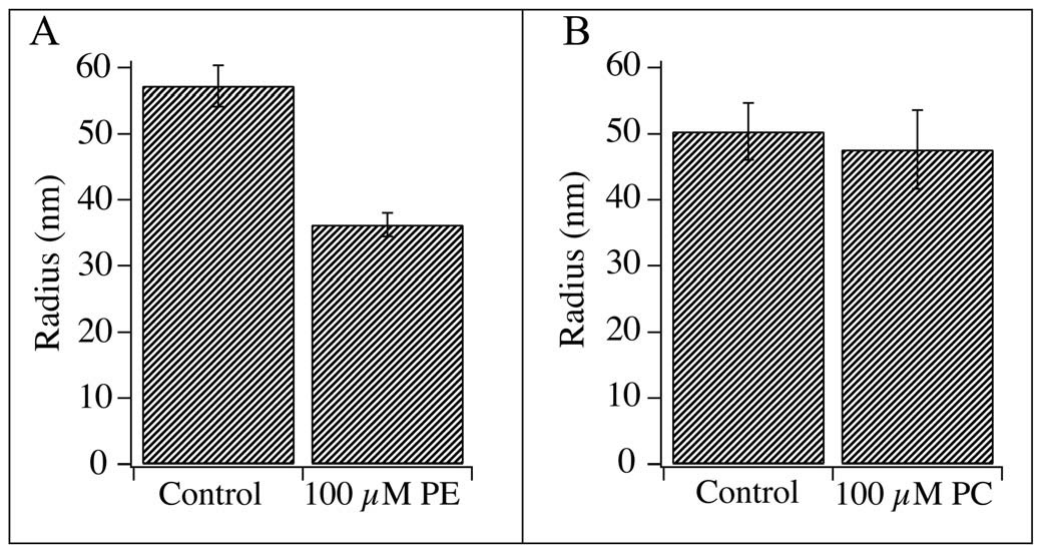
Result of asymmetric enhancements with (A) PE and (B) PC. (A) Increasing the fraction of PE in the outer leaflet of the GUV significantly decreased the nanotube radius compared to the control tube pulled from an SBL vesicle with a radius of 57±3 nm (n = 9) and 36±2 nm (n = 9) for tubes pulled from PE incubated vesicles. (B) Increasing the fraction of PC in the outer leaflet of the vesicle showed no significant effect on lipid nanotube radius with a control radius of 50±4 nm (n = 8) and a radius of 47±6 nm (n = 6) for PC incubated vesicles. Groups were tested for significant differences using a student's t-test. *** p<0.001 versus control. Results are presented as mean±SEM.

To evaluate this strategy we first measured the diameter of a lipid nanotube pulled from a GUV with pure SBL composition serving as a control measurement. Next, the distal membrane was bathed in 100 µM soy-PC or soy-PE by adding the appropriate amount of lipid to the hydration media. Following 30 min of lipid incubation, a second tube was pulled from the GUV membrane and the lipid nanotube radius was measured again. The lipids that were tested using this procedure; soy-PC and soy-PE (Figure 2 A & B) account for more than 60 percent of the total soy bean lipid extract and were chosen with the goal of maintaining the native characteristics of the system. The soy-PE was used to add negative curvature to the membrane, as the relative size of the PC and PE head groups indicate that the soy-PE has a more negative intrinsic curvature. Likewise the soy-PC was added to the lipid composition to decrease the spontaneous curvature by displacing the higher curvature soy-PE in the membrane. The bar graph in Figure 3 A shows that adding negative curvature through incubation with soy-PE resulted in a decrease in tube radii by approximately 40%. However, the nanotubes measured following soy-PC incubation showed no significant change in diameter (Figure 3 B).

Curvature-coupling is thought to direct high curvature lipids to the curved area of the membrane to optimize their packing density and decrease the effective bending rigidity. Therefore, we hypothesize that increasing or decreasing the amount of high curvature lipid available for redistribution should in part determine the nanotube diameter. To test this hypothesis, we employed a second method for symmetric manipulation of the membrane composition to control the tube diameter that consisted of altering the total lipid composition before GUV formation as illustrated in Figure 2 E & F. The lipid mixtures used to form the GUV were augmented with 10, 20, or 30% of soy-PC or soy-PE. This method has enhanced control compared to the incubation method since the absolute lipid composition is known and can easily be used to predict tube diameter. However, the dynamic aspect, meaning the ability to change the tube diameter after it is formed, is lost. It should also be mentioned that GUVs from these augmented preparations formed at a somewhat lower success rate. Indeed, the further the deviation from the original SBL composition, the less likely it was to form usable GUVs.

**Figure 4:**
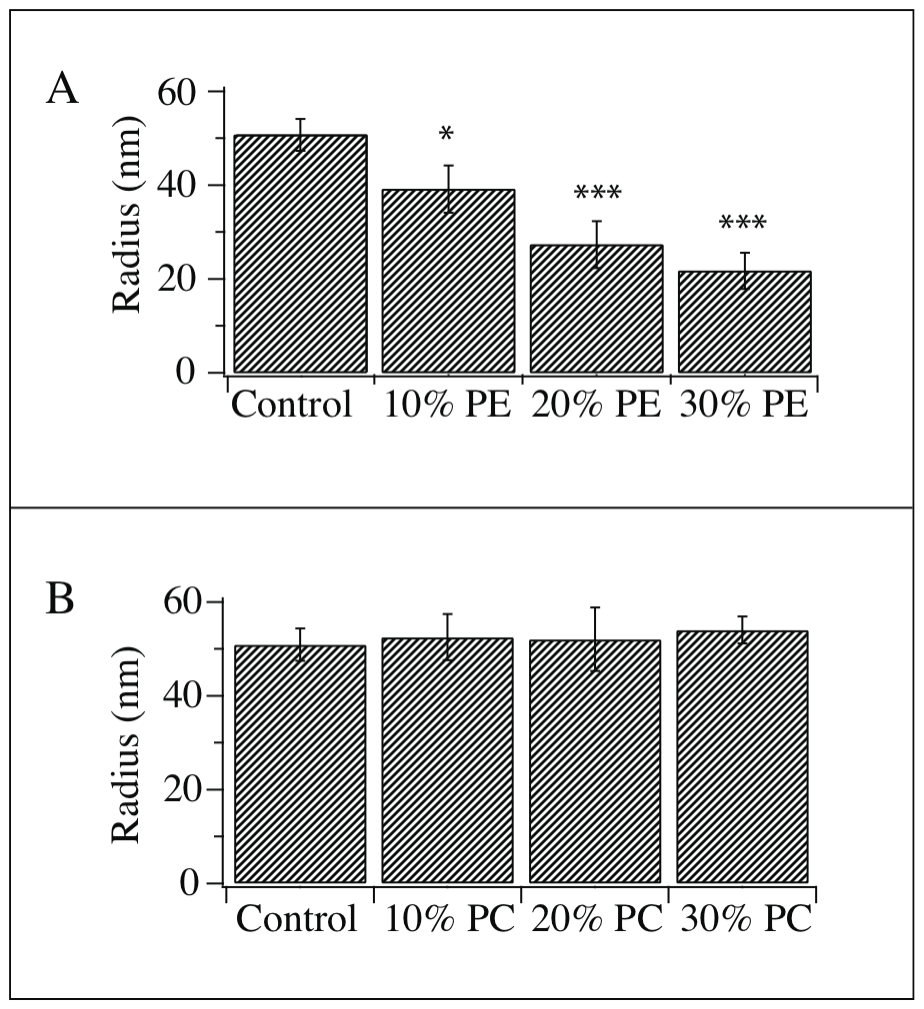
Result of symmetric enhancements with (A) PE and (B) PC. (A) The fraction of PE in the vesicle was altered by manipulating the lipid composition in the initial vesicle formation step. The nanotube radii were measured in vesicles that had been enhanced by 10, 20 and 30% PE. We found a concentration dependent constriction of the nanotube where the control radius was 51±3 nm (n = 26) and the radius of the soy-PE enhanced tubes was 39±5 nm with 10% PE (n = 7), 27±5 nm (n = 11) with 20% PE and 22±4 nm (n = 16) with 30% PE. (B) When the fraction of PC was altered with 10, 20 and 30% in the initial lipid preparation, only a very slight increase of nanotube radius was observed. The control was the same as for the PE enhancements with a radius of 51±3 nm (n = 26) while the radius of the nanotube enhanced with 10% PC was 53±5 nm (n = 8), 20% PC was 52±7 nm (n = 11) and 30% PC was 54±3 nm (n = 9). Groups were tested for significant differences using a student's t-test. * p<0.05 and *** p<0.001 versus control. Results are presented as mean±SEM.

The bar graph in Figure 4A shows the nanotube radii measured from soy-PE augmented GUVs. There is a concentration dependent constriction of the tube as the percent of soy-PE is increased (SBL = 51±3 nm, 10% PE = 39±5 nm, 20% PE = 27±5 nm, and 30% PE = 18±11 nm) indicating a decrease in the effective bending rigidity. The data from the soy-PC augmentations are shown in Figure 4B. The measurements from these nanotubes show no significant increase in tube diameter (10% PC = 53±5 nm, 20% PC = 52±7 nm and 30% PC = 54±3 nm) and indicating no change in the effective bending rigidity.

## Discussion

### Asymmetric incubation decreases nanotube diameter with soy-PE and no measurable effect with soy-PC

Similar to the lipid nanotubes that we study here, in biology, high curvature tubular membrane structures are found in cellular organelles, and in dynamic relatively short-lived membrane structures such as intracellular and extracellular transport systems [12], [13], and the lipidic fusion pore [14], [15]. For instance, membrane fusion is a ubiquitous biological process and is generally believed to advance though several intermediate membrane structures including a semi-stable fusion pore. The dynamic properties of this pore structure can be extracted from amperometric studies of exocytosis. When exocytosis is monitored with amperometry the neurochemical release from an individual fusing synaptic vesicle is translated into a current transient as the vesicle neurotransmitter content is oxidized at the electrode surface. On a subset of these events it is possible to measure a feature immediately before the spike, which often is detected as either a low current ramping or a plateau. This feature, called a pre-spike “foot” (PSF), has been attributed to the diffusion of the electro-active contents leaking through a stabilized nanometric fusion pore [16]. Hence, the duration and amplitudes of these pre-spike features reveal the activity and the stability of this lipidic pore structure.

In addition to measuring the diffusion through this structure researchers have shown that its apparent radius can be constricted by asymmetrically adding lipids of negative intrinsic curvature to the outer leaflet of the cell. For instance, Amatore et al. have shown that the foot current measured during exocytosis at chromaffin cells decreases following incubation with arachidonic acid [17] and Mellander et al. have shown that the same is true for PC12 cells incubated with phosphatidylethanolamine [18]. We have adopted this method to constrict the nanotube diameter using an outer leaflet incubation of the native soy-PE as the negative intrinsic curvature lipid. It was important to use soy lipid, as non-native lipids often impede the formation of the MLV-GUV assembly for reasons that are not currently clear.

The bar graph in figure 3A shows that following the soy-PE incubation a decrease of approximately 20 nm in nanotube radius was detected. This result suggests that the extra vesicular soy-PE is transferred into the outer leaflet of the GUV and that the intrinsic curvature of the soy-PE is negative relative to the spontaneous curvature of the SBL monolayer. The curvature of the tube is the sum of the two principle curvatures of the two dimensional surface. By definition, a cylinder has no curvature in one plane, so the inverse of the measured radius denotes the total curvature of the nanotube system. The sign is negative to indicate that the curvature is calculated for the inner diameter of the tube. The change in curvature can be calculated by subtracting the curvature (1/R) of the control measurement from the curvature of the incubated tube. Using this value (≈−1/100 nm) and assuming the intrinsic curvature of soy-PE is similar to DOPE (−1/3 nm) [19] we can estimate that the mole percent of soy-PE has increased by ≈3% in the inner leaflet of the nanotube following incubation. This estimate makes many assumptions but is illustrative in that it shows that the observed change in radius reflects a reasonable augmentation of soy-PE [20].

The fusion pore was again considered as a model when devising a method to increase nanotube diameter. It has been shown that the asymmetric incubation of the cell membrane using the positive intrinsic curvature lipid lysophosphatidylcholine (LPC) had the effect of dilating the apparent pore radius [17], [18]. There is an undetermined low amount of LPC in the SBL, therefore we opted to use soy-PC, which approaches a molecular geometry of a lamellar lipid, in an attempt to lower the mole fraction of the high curvature soy-PE in the inner leaflet of the nanotube. It has previously been shown that PC incubation can also affect exocytosis in a way which may be related to intrinsic curvature [21]. Due to the larger PC head group, the soy-PC has less negative intrinsic curvature than soy-PE although it was not clear if the intrinsic curvature would be more positive than the spontaneous curvature of the SBL monolayer.

The soy-PC incubation showed no significant change in tube diameter (Figure 3B) indicating that either the lipid did not significantly transfer into the membrane or that the spontaneous curvature after incubation was not significantly different from the spontaneous curvature of the SBL membrane. Indeed even if soy-PC was strictly lamellar (no intrinsic curvature) using the assumptions made above (3% increase and a soy-PE intrinsic curvature of −1/3 nm), the displacement of the soy-PE would amount to a very small decrease in curvature. This change in curvature would translate to an approximate increase of 6 nm in tube diameter. This is a small increase compared to the effects recorded from the soy-PE incubation, and below the error of our measurement. Furthermore it is unlikely that soy-PC has an intrinsic curvature that equals zero, due to the fact that neutral diacyl phospholipids typically have slightly negative intrinsic curvatures. This could also explain the observed lack of an increased nanotube diameter. The statistically insignificant decrease in nanotube size (3 nm difference between SBL and 100 µM PC incubation), was used to back calculate the intrinsic curvature of soy-PC based on the estimated 3% increase in soy-PC concentration and was found to be −1/8.7 nm, comparable to values reported for other phosphatidylcholines [19].

It appears that increasing the spontaneous curvature of the monolayer is not possible using this lipid for incubation. Assuming that that the lipid was enriched, it follows that the intrinsic curvature of soy-PC is not close enough to zero to effectively dilute the higher curvature elements. For this strategy to be successful it might be necessary to deviate from the major components of the SBL and use a positive intrinsic curvature lipid such soy-LPC, however preliminary attempts to incubate with 100 µM soy-LPC proved difficult in that a large cone shaped tube was formed (Figure S1) and when the concentration was reduced by 10 times we found that the tube diameter was slightly decreased (Figure S2).

### Symmetric augmentation decreases nanotube diameter with soy-PE and no measureable change with soy-PC

With the goal of better understanding how lipid composition is controlled in high curvature membrane regions, again we look for biological inspiration. Two examples, which invoke the concept of curvature-coupling, are quite instructive. The Golgi apparatus is known to be rich in sphingolipids and cholesterol, which form flat rigid bilayers. The enrichment of these lipids appears to be the result of their exclusion from budding COPI vesicles involved in recycling Golgi proteins. Two models are offered for this segregation. Either, preformed lipid domains recruit the proteins needed for vesicle budding, or the high curvature vesicular membrane formed by these proteins induces the segregation excluding the bilayer-forming lipids from the vesicles [22], [23]. Lipid sorting has also been observed in the high curvature pore structures formed between cells during mating in the protist *Tetrahymena*, where some lipids were found to be concentrated in the pore region thereby excluding others [24]. Two models were also proposed for this phenomenon, either the pores were formed from the lipid domain or the pores induced the domain. In this case further experiments showed that the lipid segregation was brought about by changes in membrane curvature [25]. Thus, if the curved components can be reorganized to facilitate curvature then the total availability of these lipids might be an indication of the bilayers potential for forming curved structures regardless of their initial distribution. Curvature-coupling was also tested with the symmetric augmentation of positively curved soy-LPC, however there was no significant change in nanotube radius (Figure S3).

The results in figure 4A show that there is an apparent relationship between the amount of soy-PE in the membrane and the effective bending rigidity. This seems to suggest that rearrangement based on curvature-coupling does take place. However, we also consider the possibility that the reduced effective bending rigidity of the membrane is the result of a destabilization, which could follow from adding such a large amount of non-lamellar lipid [26], [27]. A mechanical destabilization such as this might be ruled out by bearing in mind the incubation results; the incubation method was much more effective than the 10% total composition augmentation despite the probable low amount of lipid transfer and the fact that only half of the bilayer is in contact with the incubation solution. A softening of the membrane due to soy-PE induced destabilization would most likely be concentration dependent making the incubation less efficient. A probable explanation is that lipid asymmetry was induced in the membrane to constrict the nanotube. However there may also be an as yet undetermined effect on the tension of the system, which has not been monitored here.

The important factors for manipulating the effective bending rigidity mentioned above are the intrinsic curvature of the lipids and their concentration in the membrane. Our initial thought was that the addition of soy-PC would effectively decrease the concentration of soy-PE and thereby dilate the nanotube diameter by increasing the effective bending rigidity. However, due to the fact that no measureable dilation of the tube was detected, it appears that soy-PC instead might contribute slightly to the negative spontaneous curvature of the membrane. Therefore the addition of soy-PC seems to have a competing effect on the effective bending rigidity. The soy-PE displacement by soy-PC decreases the average intrinsic curvature of the curved species in the membrane as summarized in Table 1., however the mole fraction of lipids with negative intrinsic curvature is increased. This results in that the spontaneous curvature of the membrane has not increased enough for our method to detect the change in the effective bending rigidity. The estimated values from the asymmetric augmentations displayed in Table 1, demonstrate that increasing the abundance of soy-PC should decrease the average intrinsic curvature of the known curved elements. However, the increase in the mole fraction of negative intrinsic curvature lipids counteracts this effect leading to a zero net change in the overall spontaneous curvature for soy-PC additions. In addition to affecting the concentration of soy-PE, soy-PC augmentation also affects other lipids present in the membrane that display positive, neutral and negative spontaneous curvature. It seems that the intrinsic curvature of soy-PC does not differ enough from soy-PE to make a difference in effective bending rigidity and therefore in the case of the incubation method it will be necessary to use lipids other than soy-PC to affect the spontaneous curvature. Recently it was found, using a complimentary radius determination method, that nanotube diameter could be increased by incorporating cholesterol into the lipid preparation [7], suggesting that this membrane component might be a good candidate for future experiments.

**Table 1:** Percent of soy-PC and soy-PE and estimated contributions to monolayer spontaneous curvature for total composition additions of soy-PC and soy-PE.

	Mole fraction PC	Mole fraction PE	Average estimated intrinsic curvature PC+PE (nm^−1^)	Mole fraction PC+PE	Estimated PC+PE contribution to spontaneous curvature (nm^−1^)
30% PC	0.620	0.155	−1/6.3	0.775	−1/8
20% PC	0.566	0.177	−1/6	0.743	−1/8
10% PC	0.511	0.199	−1/5.7	0.710	−1/8
SBL	0.457	0.221	−1/5.4	0.678	−1/8
10% PE	0.411	0.299	−1/4.8	0.710	−1/7
20% PE	0.366	0.377	−1/4.4	0.743	−1/6
30% PE	0.320	0.455	−1/4.1	0.775	−1/5

The estimates in Table 1 do not reflect the spontaneous curvature of the bilayer but rather to the spontaneous curvature of the individual monolayers. If it is assumed that the membrane is symmetric, the spontaneous curvature should be zero regardless of the intrinsic curvature of added lipids. Likewise no detectable change in bending rigidity is therefore expected. The fact that increasing the spontaneous curvature of both monolayers has the effect of decreasing diameter of the nanotube indicates that addition of the intrinsic curvature lipids are being utilized to reduce the effective bending rigidity. We suggest this as evidence for curvature-coupling causing lipid asymmetry in the lipid membrane bilayers.

There is experimental evidence suggesting that lipid sorting based on intrinsic curvature is minimal in pure lipid systems [28], [29]. Molecular dynamic simulations have suggested that high curvature (radius of curvature <10 nm) is required for lipid sorting [30]. Membrane phases, however, have been shown to sense, and be segregated based on curvature [31] including lipid nanotubes [32], [33]. This behavior is attributed to the difference in bending rigidity between phases, such that the domain with reduced bending rigidity partitions into the tube allowing further constriction of the tube [33] and even leading to fission [32]. There is however at least one example of curvature-induced lipid sorting that has been demonstrated in a homogenous nanotube system [34]. This work showed that following the initial pulling of the nanotube there was a further constriction of the tube when the lipid mixture contained the negative intrinsic curvature lipid DOPE. The time scale of the constriction could be correlated to lipid diffusion and was thus attributed to the reorganization of the DOPE, which altered the effective bending rigidity in the tube region. Interestingly, the only important factors were the lipid concentration and intrinsic curvature of the added lipid. This implies that there is no minimum radius required for curvature induced lipid rearrangement and our results confirm this assertion. This suggests that the spontaneous curvature of the monolayer might be an important indicator of the potential for curvature-coupling. Furthermore, by referring back to equation 3 we find that because the system used here is under low tension [1], [5], small changes in effective bending rigidity will be reflected as large changes in radius when compared to high-tension tube measurements. Therefore curvature-coupling may be an important mechanism in these low-tension systems. This conclusion can only be drawn by assuming that the tension remains constant due to the material provided by the multi lamellar vesicle. However, because the tension is difficult to measure during these experiments, it cannot be ruled out that the tension is increased due to the addition of the high curvature lipid, possibly stress created by the opposing leaflet curvatures. Regardless of the precise mechanism for the constriction, the spontaneous curvature of the membrane appears to be the determining factor in this system.

## Conclusion

We show in the experimental results of this work that the lipid composition of the GUV is a critical factor in determining the diameter of a lipid nanotube pulled from its membrane in this model system. Here we have demonstrated two strategies based on lipid intrinsic curvature for the tailoring of lipid nanotube diameters in liposome-lipid nanotube networks. In the first approach we show that it is possible to induce lipid asymmetry by the addition of high intrinsic curvature soy-PE molecules to lower the resistance to membrane bending. The size determinations of lipid nanotubes with addition soy-PC reveal that the intrinsic curvature of soy-PC most likely is too similar to the spontaneous curvature value of the total membrane to make a measureable difference in tube diameter. We also show that increasing the amount of soy-PE in the lipid starting material decreases the lipid nanotube diameter in a concentration dependent way. Independent of whether the soy-PE is added to the outer lipid leaflet of the GUV (corresponding to the inner leaflet of the nanotube), or is added symmetrically to the lipid bilayer, the effect of constricting the lipid nanotube is recorded. These results suggest evidence of curvature-coupling, which might be an important mechanism for lipid sorting into the high curvature membranes of these low tension lipid nanotubes. Thus, to increase the diameter of the nanotube, in comparison to lipid nanotubes pulled from SBL, it seems to be necessary to expand the manipulated components beyond the two major lipids that have been investigated in this work. However there is a fair range of nanotube diameter that can be achieved using the basic system described here and from a practical stand point the amount of soy-PE is predicative of nanotube diameter.

## Supporting Information

Figure S1 **Soy-LPC incubation induced cone formation.** A differential interference contrast image of the cone formed when attempting to pull a lipid nanotube from a GUV that has been bathed in 100 µM soy-LPC.
(EPS)

Figure S2 **Result of asymmetric enhancement with Soy-LPC.** Increasing the fraction of LPC in the outer leaflet of the GUV significantly decreases the tube radius compared to the control tube pulled from an SBL vesicle with a radius of 57±3 nm (n = 3) and 44±3 nm (n = 8) For LPC incubated vesicles. * p<0.05.
(EPS)

Figure S3 **Result of symmetric enhancement with Soy-LPC.** The fraction of PE in the vesicle was altered by manipulating the lipid composition in the initial vesicle formation step. The nanotube radii were measured in vesicles that had been enhanced by 1, and 5% Soy-LPC. No significant changes were measured in the tube diameter when compared to control measurements. Control 51±3 nm (n = 26) 1% LPC 55±2 nm (n = 4) and 5% PC 58±6 nm (n = 9).
(EPS)
